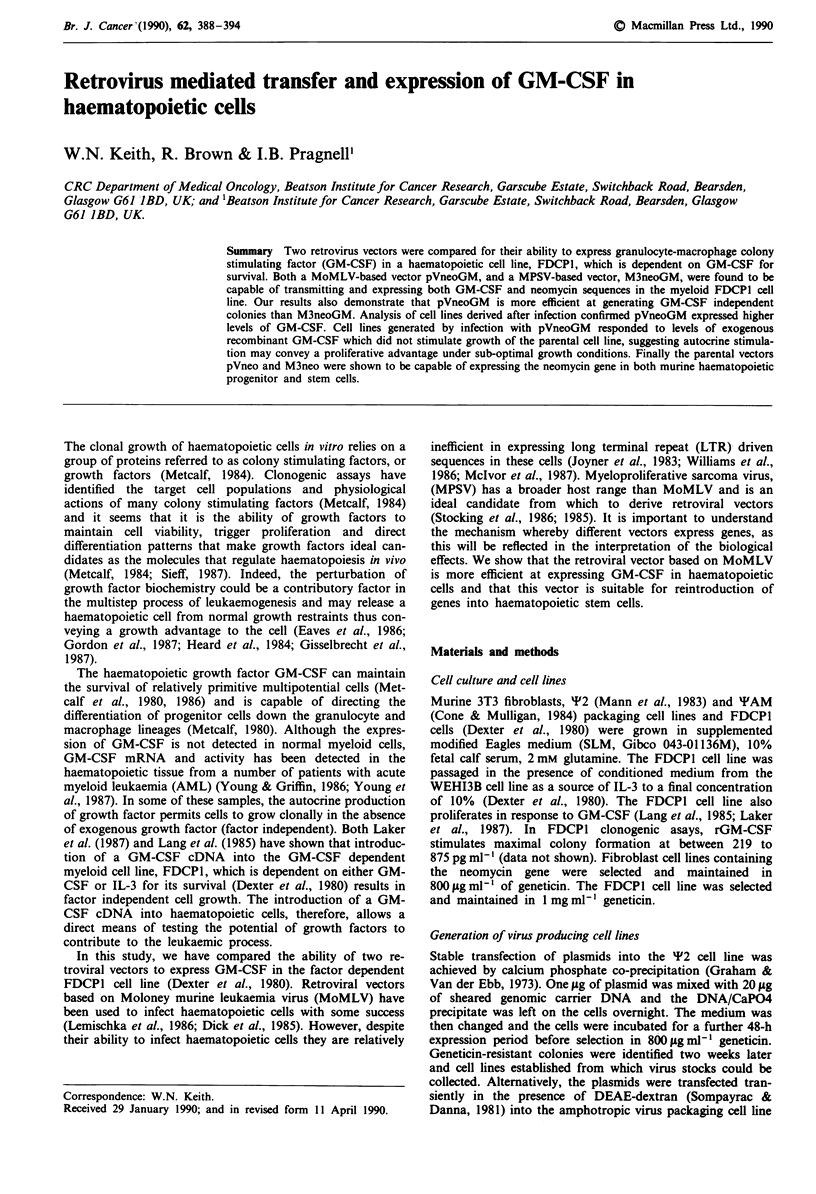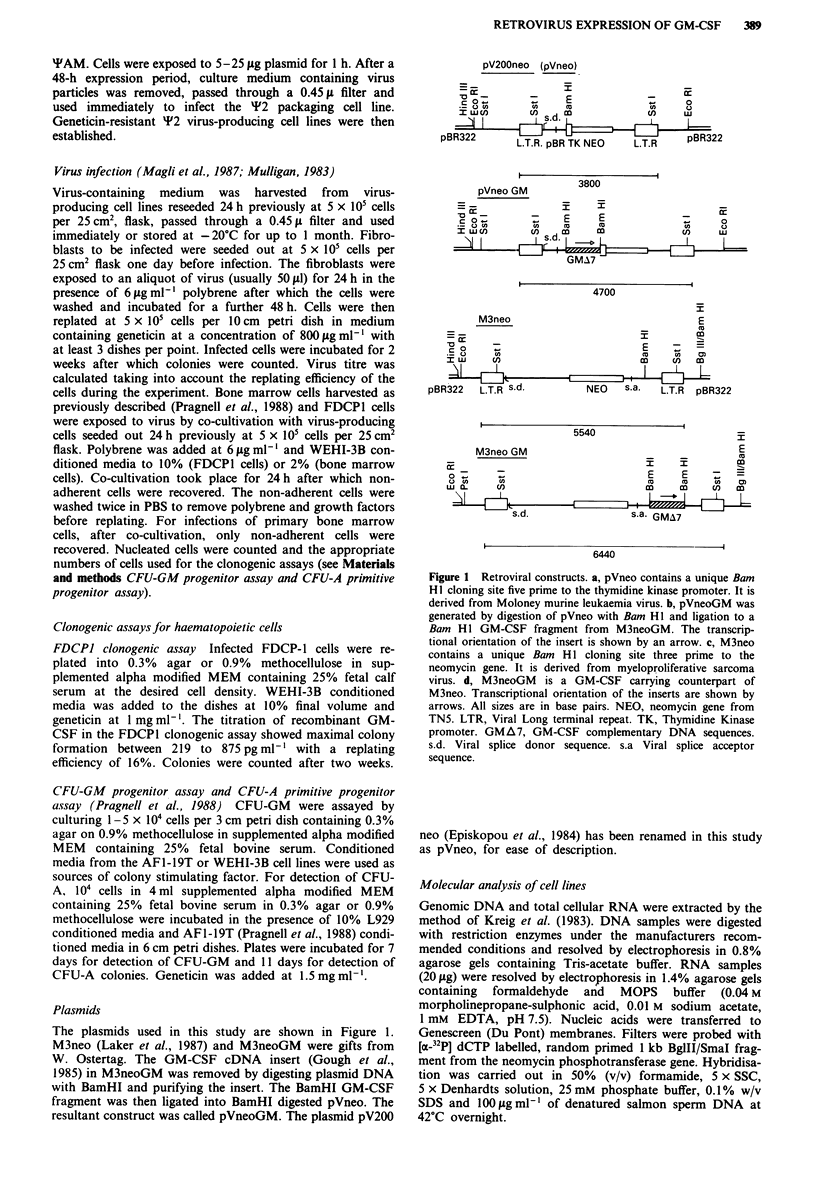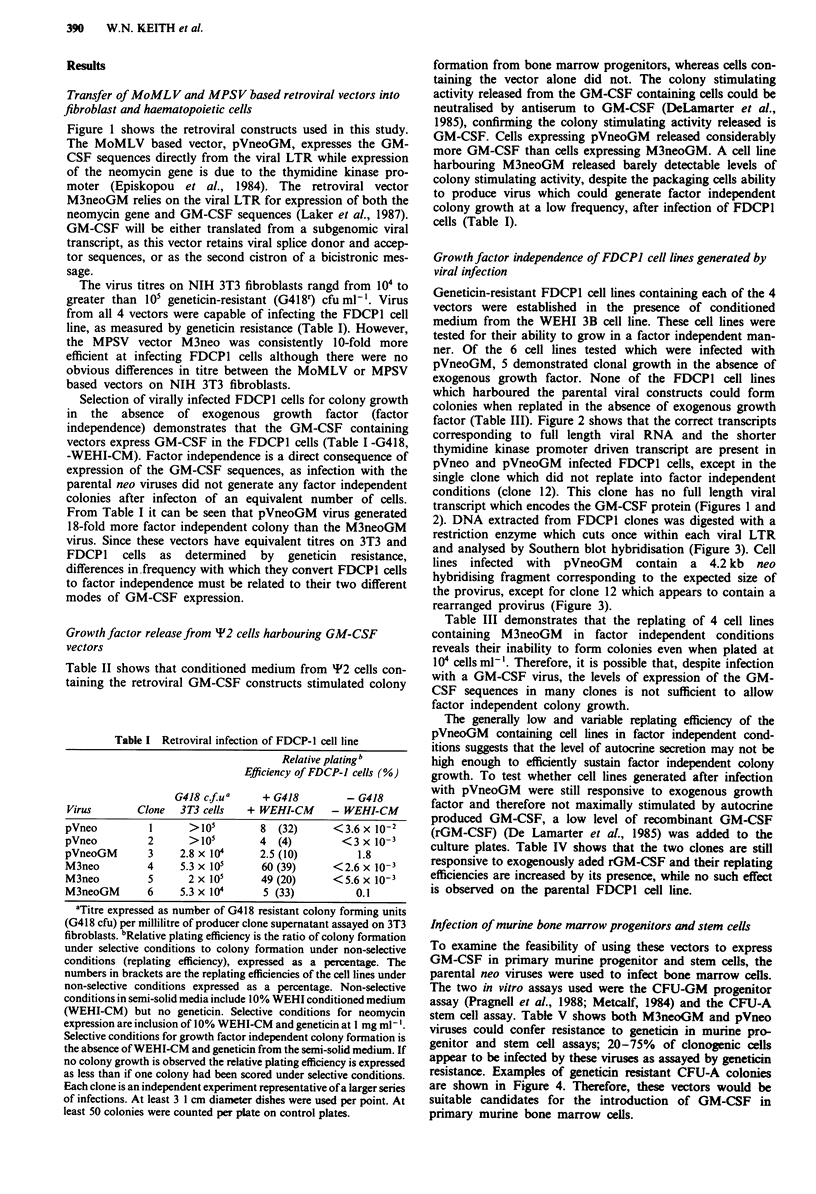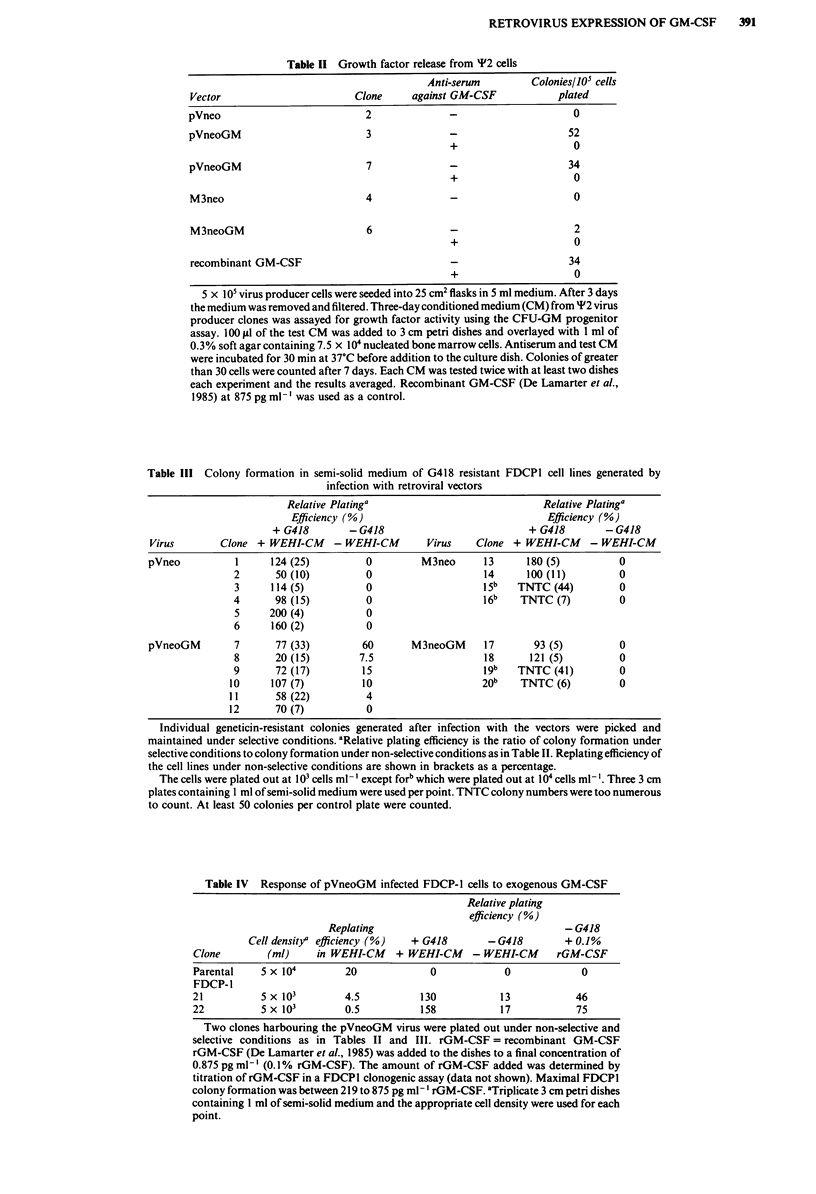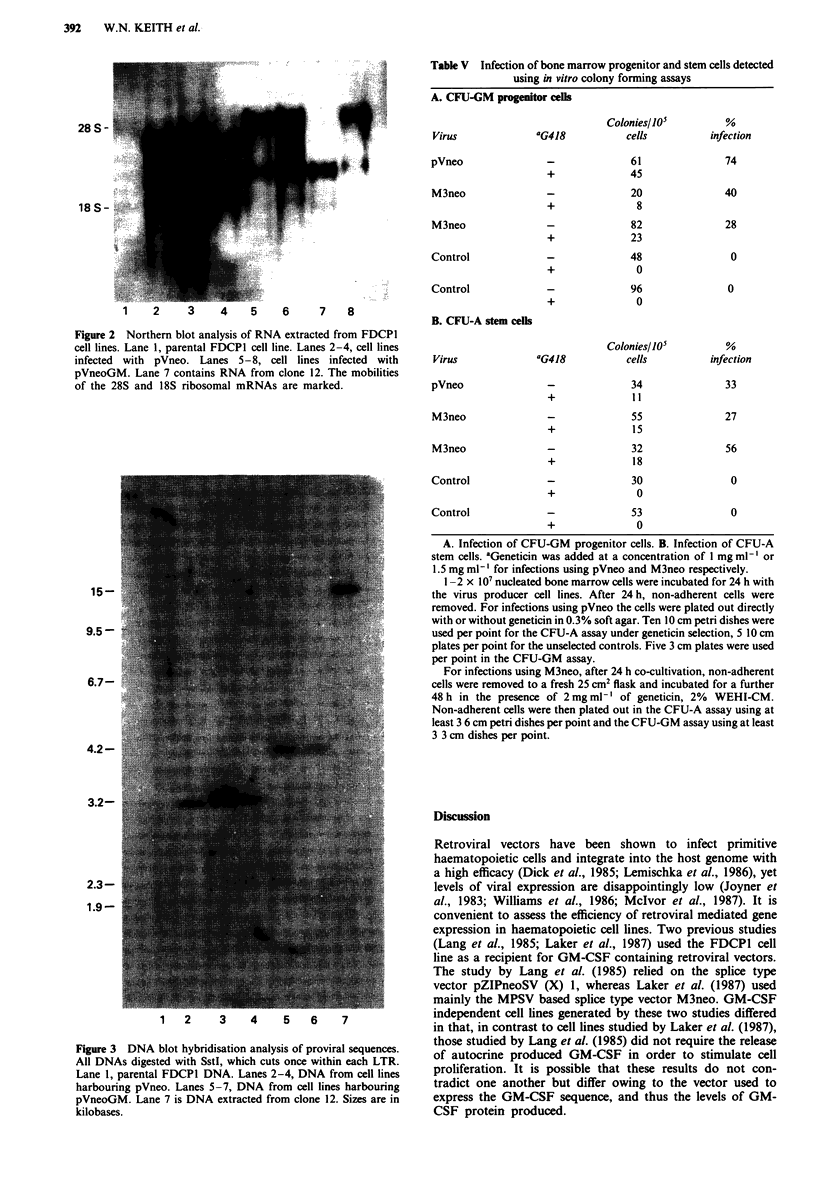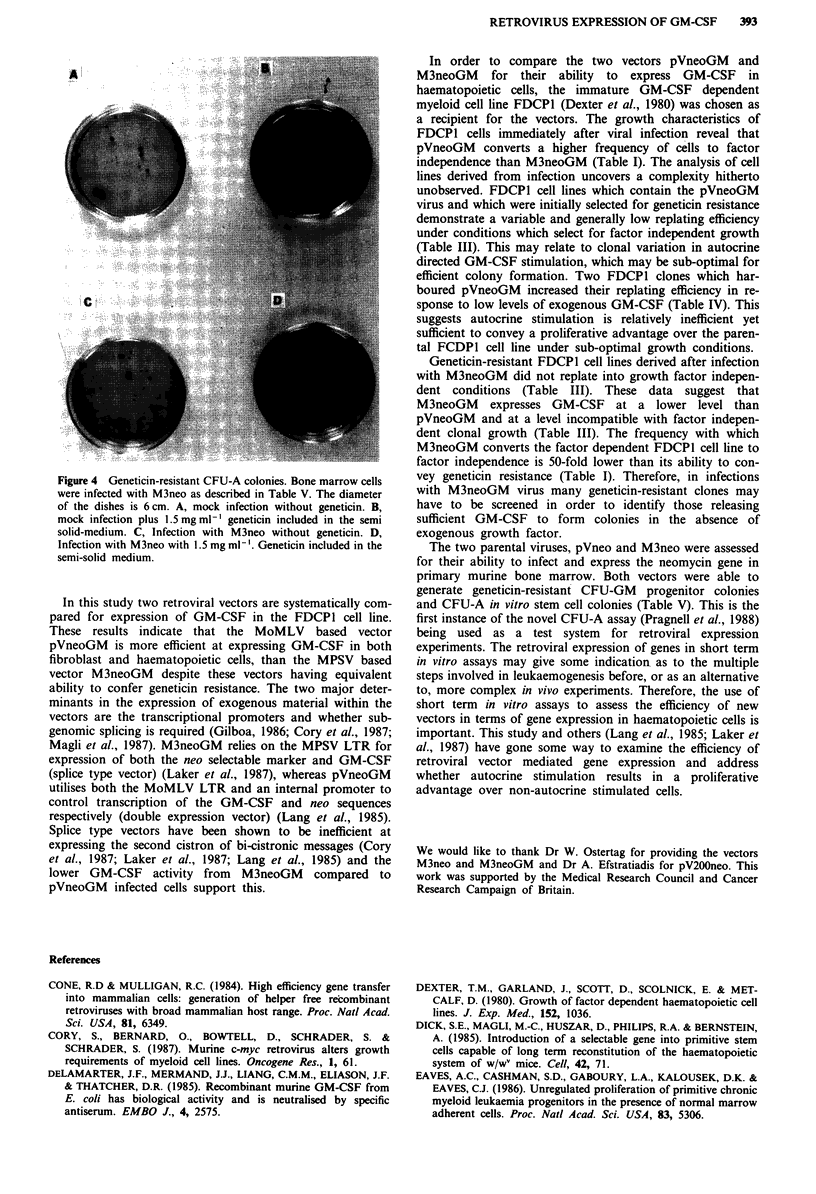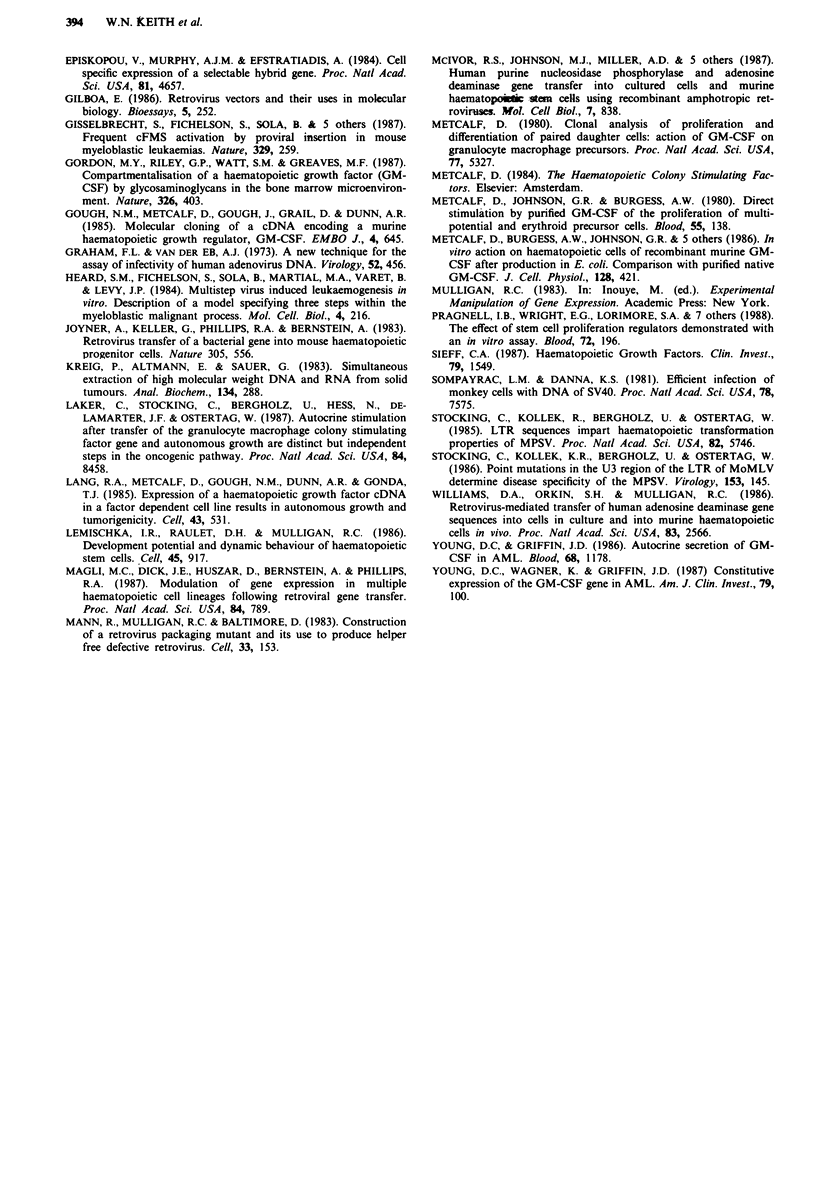# Retrovirus mediated transfer and expression of GM-CSF in haematopoietic cells.

**DOI:** 10.1038/bjc.1990.304

**Published:** 1990-09

**Authors:** W. N. Keith, R. Brown, I. B. Pragnell

**Affiliations:** CRC Department of Medical Oncology, Beatson Institute for Cancer Research, Glasgow, UK.

## Abstract

**Images:**


Br. J. Cancer (1990), 62, 388-394 © Macmillan Press Ltd., 1990

### Retrovirus mediated transfer and expression of GM-CSF in haematopoietic cells

W.N. Keith, R. Brown & I.B. Pragnell[1]

*CRC Department of Medical Oncology, Beatson Institute for Cancer Research, Garscube Estate, Switchback Road, Bearsden, Glasgow G61 1BD, UK; and [1]Beatson Institute for Cancer Research, Garscube Estate, Switchback Road, Bearsden, Glasgow G61 1BD, UK.*

**Summary** Two retrovirus vectors were compared for their ability to express granulocyte-macrophage colony stimulating factor (GM-CSF) in a haematopoietic cell line, FDCP1, which is dependent on GM-CSF for survival. Both a MoMLV-based vector pVneoGM, and a MPSV-based vector, M3neoGM, were found to be capable of transmitting and expressing both GM-CSF and neomycin sequences in the myeloid FDCP1 cell line. Our results also demonstrate that pVneoGM is more efficient at generating GM-CSF independent colonies than M3neoGM. Analysis of cell lines derived after infection confirmed pVneoGM expressed higher levels of GM-CSF. Cell lines generated by infection with pVneoGM responded to levels of exogenous recombinant GM-CSF which did not stimulate growth of the parental cell line, suggesting autocrine stimulation may convey a proliferative advantage under sub-optimal growth conditions. Finally the parental vectors pVneo and M3neo were shown to be capable of expressing the neomycin gene in both murine haematopoietic progenitor and stem cells.

The clonal growth of haematopoietic cells *in vitro* relies on a group of proteins referred to as colony stimulating factors, or growth factors (Metcalf, 1984). Clonogenic assays have identified the target cell populations and physiological actions of many colony stimulating factors (Metcalf, 1984) and it seems that it is the ability of growth factors to maintain cell viability, trigger proliferation and direct differentiation patterns that make growth factors ideal candidates as the molecules that regulate haematopoiesis *in vivo* (Metcalf, 1984; Sieff, 1987). Indeed, the perturbation of growth factor biochemistry could be a contributory factor in the multistep process of leukaemogenesis and may release a haematopoietic cell from normal growth restraints thus conveying a growth advantage to the cell (Eaves *et al.*, 1986; Gordon *et al.*, 1987; Heard *et al.*, 1984; Gisselbrecht *et al.*, 1987).

The haematopoietic growth factor GM-CSF can maintain the survival of relatively primitive multipotential cells (Metcalf *et al.*, 1980, 1986) and is capable of directing the differentiation of progenitor cells down the granulocyte and macrophage lineages (Metcalf, 1980). Although the expression of GM-CSF is not detected in normal myeloid cells, GM-CSF mRNA and activity has been detected in the haematopoietic tissue from a number of patients with acute myeloid leukaemia (AML) (Young & Griffin, 1986; Young *et al.*, 1987). In some of these samples, the autocrine production of growth factor permits cells to grow clonally in the absence of exogenous growth factor (factor independent). Both Laker *et al.* (1987) and Lang *et al.* (1985) have shown that introduction of a GM-CSF cDNA into the GM-CSF dependent myeloid cell line, FDCP1, which is dependent on either GM-CSF or IL-3 for its survival (Dexter *et al.*, 1980) results in factor independent cell growth. The introduction of a GM-CSF cDNA into haematopoietic cells, therefore, allows a direct means of testing the potential of growth factors to contribute to the leukaemic process.

In this study, we have compared the ability of two retroviral vectors to express GM-CSF in the factor dependent FDCP1 cell line (Dexter *et al.*, 1980). Retroviral vectors based on Moloney murine leukaemia virus (MoMLV) have been used to infect haematopoietic cells with some success (Lemischka *et al.*, 1986; Dick *et al.*, 1985). However, despite their ability to infect haematopoietic cells they are relatively inefficient in expressing long terminal repeat (LTR) driven sequences in these cells (Joyner *et al.*, 1983; Williams *et al.*, 1986; McIvor *et al.*, 1987). Myeloproliferative sarcoma virus, (MPSV) has a broader host range than MoMLV and is an ideal candidate from which to derive retroviral vectors (Stocking *et al.*, 1986; 1985). It is important to understand the mechanism whereby different vectors express genes, as this will be reflected in the interpretation of the biological effects. We show that the retroviral vector based on MoMLV is more efficient at expressing GM-CSF in haematopoietic cells and that this vector is suitable for reintroduction of genes into haematopoietic stem cells.

### Materials and methods

### *Cell culture and cell lines*

Murine 3T3 fibroblasts, Ψ2 (Mann *et al.*, 1983) and ΨAM (Cone & Mulligan, 1984) packaging cell lines and FDCP1 cells (Dexter *et al.*, 1980) were grown in supplemented modified Eagles medium (SLM, Gibco 043-01136M), 10% fetal calf serum, 2 mM glutamine. The FDCP1 cell line was passaged in the presence of conditioned medium from the WEHI3B cell line as a source of IL-3 to a final concentration of 10% (Dexter *et al.*, 1980). The FDCP1 cell line also proliferates in response to GM-CSF (Lang *et al.*, 1985; Laker *et al.*, 1987). In FDCP1 clonogenic asays, rGM-CSF stimulates maximal colony formation at between 219 to 875 pg $ml^{-1}$ (data not shown). Fibroblast cell lines containing the neomycin gene were selected and maintained in 800 μg $ml^{-1}$ of geneticin. The FDCP1 cell line was selected and maintained in 1 mg $ml^{-1}$ geneticin.

### *Generation of virus producing cell lines*

Stable transfection of plasmids into the Ψ2 cell line was achieved by calcium phosphate co-precipitation (Graham & Van der Ebb, 1973). One μg of plasmid was mixed with 20 μg of sheared genomic carrier DNA and the DNA/CaPO4 precipitate was left on the cells overnight. The medium was then changed and the cells were incubated for a further 48-h expression period before selection in 800 μg $ml^{-1}$ geneticin. Geneticin-resistant colonies were identified two weeks later and cell lines established from which virus stocks could be collected. Alternatively, the plasmids were transfected transiently in the presence of DEAE-dextran (Sompayrac & Danna, 1981) into the amphotropic virus packaging cell line

Correspondence: W.N. Keith.
Received 29 January 1990; and in revised form 11 April 1990.

ΨAM. Cells were exposed to 5–25 μg plasmid for 1 h. After a 48-h expression period, culture medium containing virus particles was removed, passed through a 0.45 μ filter and used immediately to infect the Ψ2 packaging cell line. Geneticin-resistant Ψ2 virus-producing cell lines were then established.

*Virus infection (Magli et al., 1987; Mulligan, 1983)*

Virus-containing medium was harvested from virus-producing cell lines reseeded 24 h previously at $5 \times 10^5$ cells per 25 cm$^2$, flask, passed through a 0.45 μ filter and used immediately or stored at −20°C for up to 1 month. Fibroblasts to be infected were seeded out at $5 \times 10^5$ cells per 25 cm$^2$ flask one day before infection. The fibroblasts were exposed to an aliquot of virus (usually 50 μl) for 24 h in the presence of 6 μg ml$^{-1}$ polybrene after which the cells were washed and incubated for a further 48 h. Cells were then replated at $5 \times 10^5$ cells per 10 cm petri dish in medium containing geneticin at a concentration of 800 μg ml$^{-1}$ with at least 3 dishes per point. Infected cells were incubated for 2 weeks after which colonies were counted. Virus titre was calculated taking into account the replating efficiency of the cells during the experiment. Bone marrow cells harvested as previously described (Pragnell *et al.*, 1988) and FDCP1 cells were exposed to virus by co-cultivation with virus-producing cells seeded out 24 h previously at $5 \times 10^5$ cells per 25 cm$^2$ flask. Polybrene was added at 6 μg ml$^{-1}$ and WEHI-3B conditioned media to 10% (FDCP1 cells) or 2% (bone marrow cells). Co-cultivation took place for 24 h after which non-adherent cells were recovered. The non-adherent cells were washed twice in PBS to remove polybrene and growth factors before replating. For infections of primary bone marrow cells, after co-cultivation, only non-adherent cells were recovered. Nucleated cells were counted and the appropriate numbers of cells used for the clonogenic assays (see **Materials and methods** *CFU-GM progenitor assay and CFU-A primitive progenitor assay*).

*Clonogenic assays for haematopoietic cells*

*FDCP1 clonogenic assay* Infected FDCP-1 cells were replated into 0.3% agar or 0.9% methocellulose in supplemented alpha modified MEM containing 25% fetal calf serum at the desired cell density. WEHI-3B conditioned media was added to the dishes at 10% final volume and geneticin at 1 mg ml$^{-1}$. The titration of recombinant GM-CSF in the FDCP1 clonogenic assay showed maximal colony formation between 219 to 875 pg ml$^{-1}$ with a replating efficiency of 16%. Colonies were counted after two weeks.

*CFU-GM progenitor assay and CFU-A primitive progenitor assay (Pragnell et al., 1988)* CFU-GM were assayed by culturing $1-5 \times 10^4$ cells per 3 cm petri dish containing 0.3% agar on 0.9% methocellulose in supplemented alpha modified MEM containing 25% fetal bovine serum. Conditioned media from the AF1-19T or WEHI-3B cell lines were used as sources of colony stimulating factor. For detection of CFU-A, $10^4$ cells in 4 ml supplemented alpha modified MEM containing 25% fetal bovine serum in 0.3% agar or 0.9% methocellulose were incubated in the presence of 10% L929 conditioned media and AF1-19T (Pragnell *et al.*, 1988) conditioned media in 6 cm petri dishes. Plates were incubated for 7 days for detection of CFU-GM and 11 days for detection of CFU-A colonies. Geneticin was added at 1.5 mg ml$^{-1}$.

*Plasmids*

The plasmids used in this study are shown in Figure 1. M3neo (Laker *et al.*, 1987) and M3neoGM were gifts from W. Ostertag. The GM-CSF cDNA insert (Gough *et al.*, 1985) in M3neoGM was removed by digesting plasmid DNA with BamHI and purifying the insert. The BamHI GM-CSF fragment was then ligated into BamHI digested pVneo. The resultant construct was called pVneoGM. The plasmid pV200

**Figure 1** Retroviral constructs. **a**, pVneo contains a unique *Bam* H1 cloning site five prime to the thymidine kinase promoter. It is derived from Moloney murine leukaemia virus. **b**, pVneoGM was generated by digestion of pVneo with *Bam* H1 and ligation to a *Bam* H1 GM-CSF fragment from M3neoGM. The transcriptional orientation of the insert is shown by an arrow. **c**, M3neo contains a unique *Bam* H1 cloning site three prime to the neomycin gene. It is derived from myeloproliferative sarcoma virus. **d**, M3neoGM is a GM-CSF carrying counterpart of M3neo. Transcriptional orientation of the inserts are shown by arrows. All sizes are in base pairs. NEO, neomycin gene from TN5. LTR, Viral Long terminal repeat. TK, Thymidine Kinase promoter. GMΔ7, GM-CSF complementary DNA sequences. s.d. Viral splice donor sequence. s.a Viral splice acceptor sequence.

neo (Episkopou *et al.*, 1984) has been renamed in this study as pVneo, for ease of description.

*Molecular analysis of cell lines*

Genomic DNA and total cellular RNA were extracted by the method of Kreig *et al.* (1983). DNA samples were digested with restriction enzymes under the manufacturers recommended conditions and resolved by electrophoresis in 0.8% agarose gels containing Tris-acetate buffer. RNA samples (20 μg) were resolved by electrophoresis in 1.4% agarose gels containing formaldehyde and MOPS buffer (0.04 M morpholinepropane-sulphonic acid, 0.01 M sodium acetate, 1 mM EDTA, pH 7.5). Nucleic acids were transferred to Genescreen (Du Pont) membranes. Filters were probed with [α-$^{32}$P] dCTP labelled, random primed 1 kb BglII/SmaI fragment from the neomycin phosphotransferase gene. Hybridisation was carried out in 50% (v/v) formamide, 5 × SSC, 5 × Denhardts solution, 25 mM phosphate buffer, 0.1% w/v SDS and 100 μg ml$^{-1}$ of denatured salmon sperm DNA at 42°C overnight.

### Results

*Transfer of MoMLV and MPSV based retroviral vectors into fibroblast and haematopoietic cells*

Figure 1 shows the retroviral constructs used in this study. The MoMLV based vector, pVneoGM, expresses the GM-CSF sequences directly from the viral LTR while expression of the neomycin gene is due to the thymidine kinase promoter (Episkopou *et al*., 1984). The retroviral vector M3neoGM relies on the viral LTR for expression of both the neomycin gene and GM-CSF sequences (Laker *et al*., 1987). GM-CSF will be either translated from a subgenomic viral transcript, as this vector retains viral splice donor and acceptor sequences, or as the second cistron of a bicistronic message.

The virus titres on NIH 3T3 fibroblasts rangd from $10^4$ to greater than $10^5$ geneticin-resistant ($G418^r$) cfu $ml^{-1}$. Virus from all 4 vectors were capable of infecting the FDCP1 cell line, as measured by geneticin resistance (Table I). However, the MPSV vector M3neo was consistently 10-fold more efficient at infecting FDCP1 cells although there were no obvious differences in titre between the MoMLV or MPSV based vectors on NIH 3T3 fibroblasts.

Selection of virally infected FDCP1 cells for colony growth in the absence of exogenous growth factor (factor independence) demonstrates that the GM-CSF containing vectors express GM-CSF in the FDCP1 cells (Table I -G418, -WEHI-CM). Factor independence is a direct consequence of expression of the GM-CSF sequences, as infection with the parental *neo* viruses did not generate any factor independent colonies after infecton of an equivalent number of cells. From Table I it can be seen that pVneoGM virus generated 18-fold more factor independent colony than the M3neoGM virus. Since these vectors have equivalent titres on 3T3 and FDCP1 cells as determined by geneticin resistance, differences in frequency with which they convert FDCP1 cells to factor independence must be related to their two different modes of GM-CSF expression.

*Growth factor release from Ψ2 cells harbouring GM-CSF vectors*

Table II shows that conditioned medium from Ψ2 cells containing the retroviral GM-CSF constructs stimulated colony formation from bone marrow progenitors, whereas cells containing the vector alone did not. The colony stimulating activity released from the GM-CSF containing cells could be neutralised by antiserum to GM-CSF (DeLamarter *et al*., 1985), confirming the colony stimulating activity released is GM-CSF. Cells expressing pVneoGM released considerably more GM-CSF than cells expressing M3neoGM. A cell line harbouring M3neoGM released barely detectable levels of colony stimulating activity, despite the packaging cells ability to produce virus which could generate factor independent colony growth at a low frequency, after infection of FDCP1 cells (Table I).

**Table I** Retroviral infection of FDCP-1 cell line

| Virus | Clone | *G418 c.f.u*[a] *3T3 cells* | *Relative plating*[b] *Efficiency of FDCP-1 cells (%)* *+ G418 + WEHI-CM* | *− G418 − WEHI-CM* |
|---|---|---|---|---|
| pVneo | 1 | $>10^5$ | 8 (32) | $<3.6\times10^{-2}$ |
| pVneo | 2 | $>10^5$ | 4 (4) | $<3\times10^{-3}$ |
| pVneoGM | 3 | $2.8\times10^4$ | 2.5 (10) | 1.8 |
| M3neo | 4 | $5.3\times10^5$ | 60 (39) | $<2.6\times10^{-3}$ |
| M3neo | 5 | $2\times10^5$ | 49 (20) | $<5.6\times10^{-3}$ |
| M3neoGM | 6 | $5.3\times10^4$ | 5 (33) | 0.1 |

[a]Titre expressed as number of G418 resistant colony forming units (G418 cfu) per millilitre of producer clone supernatant assayed on 3T3 fibroblasts. [b]Relative plating efficiency is the ratio of colony formation under selective conditions to colony formation under non-selective conditions (replating efficiency), expressed as a percentage. The numbers in brackets are the replating efficiencies of the cell lines under non-selective conditions expressed as a percentage. Non-selective conditions in semi-solid media include 10% WEHI conditioned medium (WEHI-CM) but no geneticin. Selective conditions for neomycin expression are inclusion of 10% WEHI-CM and geneticin at 1 mg $ml^{-1}$. Selective conditions for growth factor independent colony formation is the absence of WEHI-CM and geneticin from the semi-solid medium. If no colony growth is observed the relative plating efficiency is expressed as less than if one colony had been scored under selective conditions. Each clone is an independent experiment representative of a larger series of infections. At least 3 1 cm diameter dishes were used per point. At least 50 colonies were counted per plate on control plates.

*Growth factor independence of FDCP1 cell lines generated by viral infection*

Geneticin-resistant FDCP1 cell lines containing each of the 4 vectors were established in the presence of conditioned medium from the WEHI 3B cell line. These cell lines were tested for their ability to grow in a factor independent manner. Of the 6 cell lines tested which were infected with pVneoGM, 5 demonstrated clonal growth in the absence of exogenous growth factor. None of the FDCP1 cell lines which harboured the parental viral constructs could form colonies when replated in the absence of exogenous growth factor (Table III). Figure 2 shows that the correct transcripts corresponding to full length viral RNA and the shorter thymidine kinase promoter driven transcript are present in pVneo and pVneoGM infected FDCP1 cells, except in the single clone which did not replate into factor independent conditions (clone 12). This clone has no full length viral transcript which encodes the GM-CSF protein (Figures 1 and 2). DNA extracted from FDCP1 clones was digested with a restriction enzyme which cuts once within each viral LTR and analysed by Southern blot hybridisation (Figure 3). Cell lines infected with pVneoGM contain a 4.2 kb *neo* hybridising fragment corresponding to the expected size of the provirus, except for clone 12 which appears to contain a rearranged provirus (Figure 3).

Table III demonstrates that the replating of 4 cell lines containing M3neoGM in factor independent conditions reveals their inability to form colonies even when plated at $10^4$ cells $ml^{-1}$. Therefore, it is possible that, despite infection with a GM-CSF virus, the levels of expression of the GM-CSF sequences in many clones is not sufficient to allow factor independent colony growth.

The generally low and variable replating efficiency of the pVneoGM containing cell lines in factor independent conditions suggests that the level of autocrine secretion may not be high enough to efficiently sustain factor independent colony growth. To test whether cell lines generated after infection with pVneoGM were still responsive to exogenous growth factor and therefore not maximally stimulated by autocrine produced GM-CSF, a low level of recombinant GM-CSF (rGM-CSF) (De Lamarter *et al*., 1985) was added to the culture plates. Table IV shows that the two clones are still responsive to exogenously aded rGM-CSF and their replating efficiencies are increased by its presence, while no such effect is observed on the parental FDCP1 cell line.

*Infection of murine bone marrow progenitors and stem cells*

To examine the feasibility of using these vectors to express GM-CSF in primary murine progenitor and stem cells, the parental *neo* viruses were used to infect bone marrow cells. The two *in vitro* assays used were the CFU-GM progenitor assay (Pragnell *et al*., 1988; Metcalf, 1984) and the CFU-A stem cell assay. Table V shows both M3neoGM and pVneo viruses could confer resistance to geneticin in murine progenitor and stem cell assays; 20–75% of clonogenic cells appear to be infected by these viruses as assayed by geneticin resistance. Examples of geneticin resistant CFU-A colonies are shown in Figure 4. Therefore, these vectors would be suitable candidates for the introduction of GM-CSF in primary murine bone marrow cells.

**Table II** Growth factor release from Ψ2 cells

| *Vector* | *Clone* | *Anti-serum against GM-CSF* | *Colonies/$10^5$ cells plated* |
|---|---|---|---|
| pVneo | 2 | − | 0 |
| pVneoGM | 3 | − | 52 |
| | | + | 0 |
| pVneoGM | 7 | − | 34 |
| | | + | 0 |
| M3neo | 4 | − | 0 |
| M3neoGM | 6 | − | 2 |
| | | + | 0 |
| recombinant GM-CSF | | − | 34 |
| | | + | 0 |

5 × $10^5$ virus producer cells were seeded into 25 $cm^2$ flasks in 5 ml medium. After 3 days the medium was removed and filtered. Three-day conditioned medium (CM) from Ψ2 virus producer clones was assayed for growth factor activity using the CFU-GM progenitor assay. 100 μl of the test CM was added to 3 cm petri dishes and overlayed with 1 ml of 0.3% soft agar containing 7.5 × $10^4$ nucleated bone marrow cells. Antiserum and test CM were incubated for 30 min at 37°C before addition to the culture dish. Colonies of greater than 30 cells were counted after 7 days. Each CM was tested twice with at least two dishes each experiment and the results averaged. Recombinant GM-CSF (De Lamarter *et al.*, 1985) at 875 pg $ml^{-1}$ was used as a control.

**Table III** Colony formation in semi-solid medium of G418 resistant FDCP1 cell lines generated by infection with retroviral vectors

| *Virus* | *Clone* | *Relative Plating[a] Efficiency (%) + G418 + WEHI-CM* | *Relative Plating[a] Efficiency (%) − G418 − WEHI-CM* | *Virus* | *Clone* | *Relative Plating[a] Efficiency (%) + G418 + WEHI-CM* | *Relative Plating[a] Efficiency (%) − G418 − WEHI-CM* |
|---|---|---|---|---|---|---|---|
| pVneo | 1 | 124 (25) | 0 | M3neo | 13 | 180 (5) | 0 |
| | 2 | 50 (10) | 0 | | 14 | 100 (11) | 0 |
| | 3 | 114 (5) | 0 | | 15[b] | TNTC (44) | 0 |
| | 4 | 98 (15) | 0 | | 16[b] | TNTC (7) | 0 |
| | 5 | 200 (4) | 0 | | | | |
| | 6 | 160 (2) | 0 | | | | |
| pVneoGM | 7 | 77 (33) | 60 | M3neoGM | 17 | 93 (5) | 0 |
| | 8 | 20 (15) | 7.5 | | 18 | 121 (5) | 0 |
| | 9 | 72 (17) | 15 | | 19[b] | TNTC (41) | 0 |
| | 10 | 107 (7) | 10 | | 20[b] | TNTC (6) | 0 |
| | 11 | 58 (22) | 4 | | | | |
| | 12 | 70 (7) | 0 | | | | |

Individual geneticin-resistant colonies generated after infection with the vectors were picked and maintained under selective conditions. [a]Relative plating efficiency is the ratio of colony formation under selective conditions to colony formation under non-selective conditions as in Table II. Replating efficiency of the cell lines under non-selective conditions are shown in brackets as a percentage.

The cells were plated out at $10^3$ cells $ml^{-1}$ except for[b] which were plated out at $10^4$ cells $ml^{-1}$. Three 3 cm plates containing 1 ml of semi-solid medium were used per point. TNTC colony numbers were too numerous to count. At least 50 colonies per control plate were counted.

**Table IV** Response of pVneoGM infected FDCP-1 cells to exogenous GM-CSF

| *Clone* | *Cell density[a] (ml)* | *Replating efficiency (%) in WEHI-CM* | *Relative plating efficiency (%) + G418 + WEHI-CM* | *Relative plating efficiency (%) − G418 − WEHI-CM* | *Relative plating efficiency (%) − G418 + 0.1% rGM-CSF* |
|---|---|---|---|---|---|
| Parental FDCP-1 | 5 × $10^4$ | 20 | 0 | 0 | 0 |
| 21 | 5 × $10^3$ | 4.5 | 130 | 13 | 46 |
| 22 | 5 × $10^3$ | 0.5 | 158 | 17 | 75 |

Two clones harbouring the pVneoGM virus were plated out under non-selective and selective conditions as in Tables II and III. rGM-CSF = recombinant GM-CSF rGM-CSF (De Lamarter *et al.*, 1985) was added to the dishes to a final concentration of 0.875 pg $ml^{-1}$ (0.1% rGM-CSF). The amount of rGM-CSF added was determined by titration of rGM-CSF in a FDCP1 clonogenic assay (data not shown). Maximal FDCP1 colony formation was between 219 to 875 pg $ml^{-1}$ rGM-CSF. [a]Triplicate 3 cm petri dishes containing 1 ml of semi-solid medium and the appropriate cell density were used for each point.

**Figure 2** Northern blot analysis of RNA extracted from FDCP1 cell lines. Lane 1, parental FDCP1 cell line. Lanes 2–4, cell lines infected with pVneo. Lanes 5–8, cell lines infected with pVneoGM. Lane 7 contains RNA from clone 12. The mobilities of the 28S and 18S ribosomal mRNAs are marked.

**Figure 3** DNA blot hybridisation analysis of proviral sequences. All DNAs digested with SstI, which cuts once within each LTR. Lane 1, parental FDCP1 DNA. Lanes 2–4, DNA from cell lines harbouring pVneo. Lanes 5–7, DNA from cell lines harbouring pVneoGM. Lane 7 is DNA extracted from clone 12. Sizes are in kilobases.

**Table V** Infection of bone marrow progenitor and stem cells detected using *in vitro* colony forming assays

| *Virus* | [a]*G418* | *Colonies/$10^5$ cells* | *% infection* |
|---|---|---|---|
| **A. CFU-GM progenitor cells** | | | |
| pVneo | – | 61 | 74 |
| | + | 45 | |
| M3neo | – | 20 | 40 |
| | + | 8 | |
| M3neo | – | 82 | 28 |
| | + | 23 | |
| Control | – | 48 | 0 |
| | + | 0 | |
| Control | – | 96 | 0 |
| | + | 0 | |
| **B. CFU-A stem cells** | | | |
| pVneo | – | 34 | 33 |
| | + | 11 | |
| M3neo | – | 55 | 27 |
| | + | 15 | |
| M3neo | – | 32 | 56 |
| | + | 18 | |
| Control | – | 30 | 0 |
| | + | 0 | |
| Control | – | 53 | 0 |
| | + | 0 | |

A. Infection of CFU-GM progenitor cells. B. Infection of CFU-A stem cells. [a]Geneticin was added at a concentration of 1 mg $ml^{-1}$ or 1.5 mg $ml^{-1}$ for infections using pVneo and M3neo respectively.

1–2 × $10^7$ nucleated bone marrow cells were incubated for 24 h with the virus producer cell lines. After 24 h, non-adherent cells were removed. For infections using pVneo the cells were plated out directly with or without geneticin in 0.3% soft agar. Ten 10 cm petri dishes were used per point for the CFU-A assay under geneticin selection, 5 10 cm plates per point for the unselected controls. Five 3 cm plates were used per point in the CFU-GM assay.

For infections using M3neo, after 24 h co-cultivation, non-adherent cells were removed to a fresh 25 $cm^2$ flask and incubated for a further 48 h in the presence of 2 mg $ml^{-1}$ of geneticin, 2% WEHI-CM. Non-adherent cells were then plated out in the CFU-A assay using at least 3 6 cm petri dishes per point and the CFU-GM assay using at least 3 3 cm dishes per point.

### Discussion

Retroviral vectors have been shown to infect primitive haematopoietic cells and integrate into the host genome with a high efficacy (Dick *et al.*, 1985; Lemischka *et al.*, 1986), yet levels of viral expression are disappointingly low (Joyner *et al.*, 1983; Williams *et al.*, 1986; McIvor *et al.*, 1987). It is convenient to assess the efficiency of retroviral mediated gene expression in haematopoietic cell lines. Two previous studies (Lang *et al.*, 1985; Laker *et al.*, 1987) used the FDCP1 cell line as a recipient for GM-CSF containing retroviral vectors. The study by Lang *et al.* (1985) relied on the splice type vector pZIPneoSV (X) 1, whereas Laker *et al.* (1987) used mainly the MPSV based splice type vector M3neo. GM-CSF independent cell lines generated by these two studies differed in that, in contrast to cell lines studied by Laker *et al.* (1987), those studied by Lang *et al.* (1985) did not require the release of autocrine produced GM-CSF in order to stimulate cell proliferation. It is possible that these results do not contradict one another but differ owing to the vector used to express the GM-CSF sequence, and thus the levels of GM-CSF protein produced.

**Figure 4** Geneticin-resistant CFU-A colonies. Bone marrow cells were infected with M3neo as described in Table V. The diameter of the dishes is 6 cm. A, mock infection without geneticin. B, mock infection plus 1.5 mg $ml^{-1}$ geneticin included in the semi solid-medium. C, Infection with M3neo without geneticin. D, Infection with M3neo with 1.5 mg $ml^{-1}$. Geneticin included in the semi-solid medium.

In this study two retroviral vectors are systematically compared for expression of GM-CSF in the FDCP1 cell line. These results indicate that the MoMLV based vector pVneoGM is more efficient at expressing GM-CSF in both fibroblast and haematopoietic cells, than the MPSV based vector M3neoGM despite these vectors having equivalent ability to confer geneticin resistance. The two major determinants in the expression of exogenous material within the vectors are the transcriptional promoters and whether subgenomic splicing is required (Gilboa, 1986; Cory *et al.*, 1987; Magli *et al.*, 1987). M3neoGM relies on the MPSV LTR for expression of both the *neo* selectable marker and GM-CSF (splice type vector) (Laker *et al.*, 1987), whereas pVneoGM utilises both the MoMLV LTR and an internal promoter to control transcription of the GM-CSF and *neo* sequences respectively (double expression vector) (Lang *et al.*, 1985). Splice type vectors have been shown to be inefficient at expressing the second cistron of bi-cistronic messages (Cory *et al.*, 1987; Laker *et al.*, 1987; Lang *et al.*, 1985) and the lower GM-CSF activity from M3neoGM compared to pVneoGM infected cells support this.

In order to compare the two vectors pVneoGM and M3neoGM for their ability to express GM-CSF in haematopoietic cells, the immature GM-CSF dependent myeloid cell line FDCP1 (Dexter *et al.*, 1980) was chosen as a recipient for the vectors. The growth characteristics of FDCP1 cells immediately after viral infection reveal that pVneoGM converts a higher frequency of cells to factor independence than M3neoGM (Table I). The analysis of cell lines derived from infection uncovers a complexity hitherto unobserved. FDCP1 cell lines which contain the pVneoGM virus and which were initially selected for geneticin resistance demonstrate a variable and generally low replating efficiency under conditions which select for factor independent growth (Table III). This may relate to clonal variation in autocrine directed GM-CSF stimulation, which may be sub-optimal for efficient colony formation. Two FDCP1 clones which harboured pVneoGM increased their replating efficiency in response to low levels of exogenous GM-CSF (Table IV). This suggests autocrine stimulation is relatively inefficient yet sufficient to convey a proliferative advantage over the parental FCDP1 cell line under sub-optimal growth conditions.

Geneticin-resistant FDCP1 cell lines derived after infection with M3neoGM did not replate into growth factor independent conditions (Table III). These data suggest that M3neoGM expresses GM-CSF at a lower level than pVneoGM and at a level incompatible with factor independent clonal growth (Table III). The frequency with which M3neoGM converts the factor dependent FDCP1 cell line to factor independence is 50-fold lower than its ability to convey geneticin resistance (Table I). Therefore, in infections with M3neoGM virus many geneticin-resistant clones may have to be screened in order to identify those releasing sufficient GM-CSF to form colonies in the absence of exogenous growth factor.

The two parental viruses, pVneo and M3neo were assessed for their ability to infect and express the neomycin gene in primary murine bone marrow. Both vectors were able to generate geneticin-resistant CFU-GM progenitor colonies and CFU-A *in vitro* stem cell colonies (Table V). This is the first instance of the novel CFU-A assay (Pragnell *et al.*, 1988) being used as a test system for retroviral expression experiments. The retroviral expression of genes in short term *in vitro* assays may give some indication as to the multiple steps involved in leukaemogenesis before, or as an alternative to, more complex *in vivo* experiments. Therefore, the use of short term *in vitro* assays to assess the efficiency of new vectors in terms of gene expression in haematopoietic cells is important. This study and others (Lang *et al.*, 1985; Laker *et al.*, 1987) have gone some way to examine the efficiency of retroviral vector mediated gene expression and address whether autocrine stimulation results in a proliferative advantage over non-autocrine stimulated cells.

We would like to thank Dr W. Ostertag for providing the vectors M3neo and M3neoGM and Dr A. Efstratiadis for pV200neo. This work was supported by the Medical Research Council and Cancer Research Campaign of Britain.

**References**

CONE, R.D & MULLIGAN, R.C. (1984). High efficiency gene transfer into mammalian cells: generation of helper free recombinant retroviruses with broad mammalian host range. *Proc. Natl Acad. Sci. USA*, **81**, 6349.

CORY, S., BERNARD, O., BOWTELL, D., SCHRADER, S. & SCHRADER, S. (1987). Murine c-*myc* retrovirus alters growth requirements of myeloid cell lines. *Oncogene Res.*, **1**, 61.

DELAMARTER, J.F., MERMAND, J.J., LIANG, C.M.M., ELIASON, J.F. & THATCHER, D.R. (1985). Recombinant murine GM-CSF from *E. coli* has biological activity and is neutralised by specific antiserum. *EMBO J.*, **4**, 2575.

DEXTER, T.M., GARLAND, J., SCOTT, D., SCOLNICK, E. & METCALF, D. (1980). Growth of factor dependent haematopoietic cell lines. *J. Exp. Med.*, **152**, 1036.

DICK, S.E., MAGLI, M.-C., HUSZAR, D., PHILIPS, R.A. & BERNSTEIN, A. (1985). Introduction of a selectable gene into primitive stem cells capable of long term reconstitution of the haematopoietic system of w/$w^v$ mice. *Cell*, **42**, 71.

EAVES, A.C., CASHMAN, S.D., GABOURY, L.A., KALOUSEK, D.K. & EAVES, C.J. (1986). Unregulated proliferation of primitive chronic myeloid leukaemia progenitors in the presence of normal marrow adherent cells. *Proc. Natl Acad. Sci. USA*, **83**, 5306.

EPISKOPOU, V., MURPHY, A.J.M. & EFSTRATIADIS, A. (1984). Cell specific expression of a selectable hybrid gene. *Proc. Natl Acad. Sci. USA*, **81**, 4657.

GILBOA, E. (1986). Retrovirus vectors and their uses in molecular biology. *Bioessays*, **5**, 252.

GISSELBRECHT, S., FICHELSON, S., SOLA, B. & 5 others (1987). Frequent cFMS activation by proviral insertion in mouse myeloblastic leukaemias. *Nature*, **329**, 259.

GORDON, M.Y., RILEY, G.P., WATT, S.M. & GREAVES, M.F. (1987). Compartmentalisation of a haematopoietic growth factor (GM-CSF) by glycosaminoglycans in the bone marrow microenvironment. *Nature*, **326**, 403.

GOUGH, N.M., METCALF, D., GOUGH, J., GRAIL, D. & DUNN, A.R. (1985). Molecular cloning of a cDNA encoding a murine haematopoietic growth regulator, GM-CSF. *EMBO J.*, **4**, 645.

GRAHAM, F.L. & VAN DER EB, A.J. (1973). A new technique for the assay of infectivity of human adenovirus DNA. *Virology*, **52**, 456.

HEARD, S.M., FICHELSON, S., SOLA, B., MARTIAL, M.A., VARET, B. & LEVY, J.P. (1984). Multistep virus induced leukaemogenesis *in vitro*. Description of a model specifying three steps within the myeloblastic malignant process. *Mol. Cell. Biol.*, **4**, 216.

JOYNER, A., KELLER, G., PHILLIPS, R.A. & BERNSTEIN, A. (1983). Retrovirus transfer of a bacterial gene into mouse haematopoietic progenitor cells. *Nature* 305, 556.

KREIG, P., ALTMANN, E. & SAUER, G. (1983). Simultaneous extraction of high molecular weight DNA and RNA from solid tumours. *Anal. Biochem.*, **134**, 288.

LAKER, C., STOCKING, C., BERGHOLZ, U., HESS, N., DE-LAMARTER, J.F. & OSTERTAG, W. (1987). Autocrine stimulation after transfer of the granulocyte macrophage colony stimulating factor gene and autonomous growth are distinct but independent steps in the oncogenic pathway. *Proc. Natl Acad. Sci. USA*, **84**, 8458.

LANG, R.A., METCALF, D., GOUGH, N.M., DUNN, A.R. & GONDA, T.J. (1985). Expression of a haematopoietic growth factor cDNA in a factor dependent cell line results in autonomous growth and tumorigenicity. *Cell*, **43**, 531.

LEMISCHKA, I.R., RAULET, D.H. & MULLIGAN, R.C. (1986). Development potential and dynamic behaviour of haematopoietic stem cells. *Cell*, **45**, 917.

MAGLI, M.C., DICK, J.E., HUSZAR, D., BERNSTEIN, A. & PHILLIPS, R.A. (1987). Modulation of gene expression in multiple haematopoietic cell lineages following retroviral gene transfer. *Proc. Natl Acad. Sci. USA*, **84**, 789.

MANN, R., MULLIGAN, R.C. & BALTIMORE, D. (1983). Construction of a retrovirus packaging mutant and its use to produce helper free defective retrovirus. *Cell*, **33**, 153.

MCIVOR, R.S., JOHNSON, M.J., MILLER, A.D. & 5 others (1987). Human purine nucleosidase phosphorylase and adenosine deaminase gene transfer into cultured cells and murine haematopoietic stem cells using recombinant amphotropic retroviruses. *Mol. Cell Biol.*, **7**, 838.

METCALF, D. (1980). Clonal analysis of proliferation and differentiation of paired daughter cells: action of GM-CSF on granulocyte macrophage precursors. *Proc. Natl Acad. Sci. USA*, **77**, 5327.

METCALF, D. (1984). *The Haematopoietic Colony Stimulating Factors*. Elsevier: Amsterdam.

METCALF, D., JOHNSON, G.R. & BURGESS, A.W. (1980). Direct stimulation by purified GM-CSF of the proliferation of multipotential and erythroid precursor cells. *Blood*, **55**, 138.

METCALF, D., BURGESS, A.W., JOHNSON, G.R. & 5 others (1986). *In vitro* action on haematopoietic cells of recombinant murine GM-CSF after production in *E. coli*. Comparison with purified native GM-CSF. *J. Cell. Physiol.*, **128**, 421.

MULLIGAN, R.C. (1983). In: Inouye, M. (ed.). *Experimental Manipulation of Gene Expression*. Academic Press: New York.

PRAGNELL, I.B., WRIGHT, E.G., LORIMORE, S.A. & 7 others (1988). The effect of stem cell proliferation regulators demonstrated with an *in vitro* assay. *Blood*, **72**, 196.

SIEFF, C.A. (1987). Haematopoietic Growth Factors. *Clin. Invest.*, **79**, 1549.

SOMPAYRAC, L.M. & DANNA, K.S. (1981). Efficient infection of monkey cells with DNA of SV40. *Proc. Natl Acad. Sci. USA*, **78**, 7575.

STOCKING, C., KOLLEK, R., BERGHOLZ, U. & OSTERTAG, W. (1985). LTR sequences impart haematopoietic transformation properties of MPSV. *Proc. Natl Acad. Sci. USA*, **82**, 5746.

STOCKING, C., KOLLEK, K.R., BERGHOLZ, U. & OSTERTAG, W. (1986). Point mutations in the U3 region of the LTR of MoMLV determine disease specificity of the MPSV. *Virology*, **153**, 145.

WILLIAMS, D.A., ORKIN, S.H. & MULLIGAN, R.C. (1986). Retrovirus-mediated transfer of human adenosine deaminase gene sequences into cells in culture and into murine haematopoietic cells *in vivo*. *Proc. Natl Acad. Sci. USA*, **83**, 2566.

YOUNG, D.C, & GRIFFIN, J.D. (1986). Autocrine secretion of GM-CSF in AML. *Blood*, **68**, 1178.

YOUNG, D.C., WAGNER, K. & GRIFFIN, J.D. (1987) Constitutive expression of the GM-CSF gene in AML. *Am. J. Clin. Invest.*, **79**, 100.